# A protein chaperone can stabilize the intein‐containing precursor to indirectly promote protein splicing

**DOI:** 10.1002/pro.70768

**Published:** 2026-08-14

**Authors:** John S. Smetana, Christopher R. Powell, Noy Bagdadi, Tia M. Ariagno, Hazel Thomas, Joel Weinberger, Eyal Gur, Christopher W. Lennon

**Affiliations:** ^1^ Department of Biological Sciences Murray State University Murray Kentucky USA; ^2^ Department of Life Sciences Ben‐Gurion University of the Negev Beer‐Sheva Israel

**Keywords:** chaperone, DnaB, GroEL, GroES, intein, *Mycobacterium smegmatis*, protein engineering, protein splicing

## Abstract

*In*tervening pro*teins* (inteins) interrupt host protein sequences and are removed by a self‐mediated protein splicing reaction. Inteins are abundant in the microbial world and are often found within essential genes involved in DNA replication, recombination, and repair. Compelling examples of conditional protein splicing, where intein removal and subsequent host protein activation are highly dependent on environmental cues, suggest that some inteins serve as post‐translational regulatory elements. Within the cellular context, however, the factors influencing protein splicing are still largely unknown. In this work, we demonstrate that the splicing of an intein from *Mycobacterium smegmatis* DnaB, an essential helicase, is temperature sensitive. Using two artificial extein systems—a *k*anamycin *i*ntein *s*plicing *r*eporter (KISR) and an in vitro splicing reporter—we show that splicing is inhibited at elevated, yet physiological, temperatures. In accordance with our results from *M. smegmatis*, we find that overexpression of GroEL in *Escherichia coli* expressing the KISR system drastically increased antibiotic resistance in a temperature‐dependent manner. Under heat stress, we find that GroEL dramatically increases the levels of unspliced precursor protein. Mechanistically, GroEL does not appear to directly promote protein splicing, but rather increases the pool of unspliced precursor, which in turn results in more overall splicing. These findings represent the first description of a chaperone promoting protein splicing, demonstrating the ability of cellular factors to influence intein excision. From a physiological perspective, we hypothesize that a *M. smegmatis* DnaB intein may act as a switch to inhibit DNA replication under conditions of heat stress. Extending past this, our findings have broad implications for improving the efficiency of intein‐based protein engineering technologies.

## 1 INTRODUCTION

*In*tervening pro*teins* (inteins) are mobile genetic elements that are translated within the protein‐coding sequences of their host organisms and removed through protein splicing (Mills et al., 2014; Wood et al., 2023). In this reaction, the intein segment excises itself from the flanking sequences, termed the N‐ and C‐exteins, in a self‐mediated reaction to form the mature host protein. From an application standpoint, this unique ability to reliably and controllably rearrange peptide bonds has inspired considerable interest in the protein engineering community, leading to dozens of intein‐based technologies (Sarmiento & Camarero, 2019; Wood et al., 2023; Wood & Camarero, 2014).

From an application standpoint, this unique ability to reliably and controllably rearrange peptide bonds has inspired considerable interest in the protein engineering community, leading to dozens of intein‐based technologies. These include, but by no means are limited to, scaffolds for bioseparations, peptide cyclization, biosensor development, segmental isotope labeling, protein semisynthesis, and regulation of protein function in vivo (Mourenza et al., 2023; Perler & Allewell, 2014; Prabhala et al., 2022; Sarmiento & Camarero, 2019; Shah & Muir, 2014; Wood et al., 2023; Wood & Camarero, 2014). Exciting novel techniques continue to be regularly developed, including use of inteins for adeno‐associated virus‐mediated gene delivery for disease treatment, as well as programmable in vivo protein editing (Beyer et al., 2025; Ferla et al., 2025; Hua et al., 2025; Tasfaout et al., 2024; Zhou et al., 2024). As inteins have unique properties that have proven useful time and time again, it is hardly hyperbole that the biotechnological potential of these elements remains vast.

While inteins have proven especially useful for a variety of engineering applications, the biological roles of inteins have remained elusive for decades. Due to the role of many inteins as mobile genetic elements, these sequences were generally thought to be nothing more than selfish molecular parasites (Gogarten & Hilario, 2006; Naor et al., 2016). In contrast to this traditional view, compelling examples of conditional protein splicing (CPS) have emerged, whereby the rate and accuracy of intein excision vary greatly in response to environmental cues such as temperature, oxidative stress, salt, and even DNA damage (Belfort, 2017; Callahan et al., 2011; Ciragan et al., 2016; Green et al., 2019; Kelley et al., 2018; Lennon et al., 2016, 2018, 2019, 2021; Lennon & Belfort, 2017; Mills & Paulus, 2001; Reitter et al., 2016; Topilina, Green, et al., 2015; Topilina, Novikova, et al., 2015; Wood et al., 2023, 2020; Yalala et al., 2022). This body of evidence suggests that some inteins have evolved over time from being parasites to now acting as post‐translational regulatory elements, allowing host organisms to exert greater control over intein‐containing proteins in response to stress. Recent work has examined CPS in vivo in both archaeal and bacterial systems (Liman et al., 2024; Schneider et al., 2024), suggesting that intein‐mediated regulation may be a common, albeit underappreciated, regulatory strategy in the microbial world. Originally, CPS was a term coined for the protein engineering (Mootz et al., 2003), but was later adopted to also describe intein‐based post‐translational regulation in nature. In the engineering sense, CPS is the control the protein splicing reaction as a means to regulate the activity of the intein‐containing protein, using either contiguous or split inteins, by a variety of triggers including small molecules, light, and proteases (Di Ventura & Mootz, 2019).

Inteins are found in about half of archaeal and a quarter of bacterial genomes (Novikova et al., 2014, 2016). While abundant and widespread, inteins are not randomly distributed in microbial genomes (Novikova et al., 2014, 2016). Rather, about two‐thirds of inteins reside in proteins involved in DNA replication, recombination, and repair (RRR proteins). Additionally, approximately 70% of known inteins map to the ATP‐binding domains of ATPases. Remarkably, inteins have been retained in non‐orthologous proteins of homologous function. For example, the most common intein‐containing proteins in their respective domains are MCM in archaea and DnaB in bacteria (Novikova et al., 2016). While both MCM and DnaB are DNA helicases essential for replication, they evolved independently. The conserved clustering in RRR proteins suggests that these organisms benefit from inteins acting as post‐translational regulators to control DNA transactions. As inteins are absent from humans, they represent novel antimicrobial targets (Chan et al., 2016; Li et al., 2019, 2021; Tharappel et al., 2022; Wall et al., 2021; Zhang et al., 2011).

**FIGURE 1:**
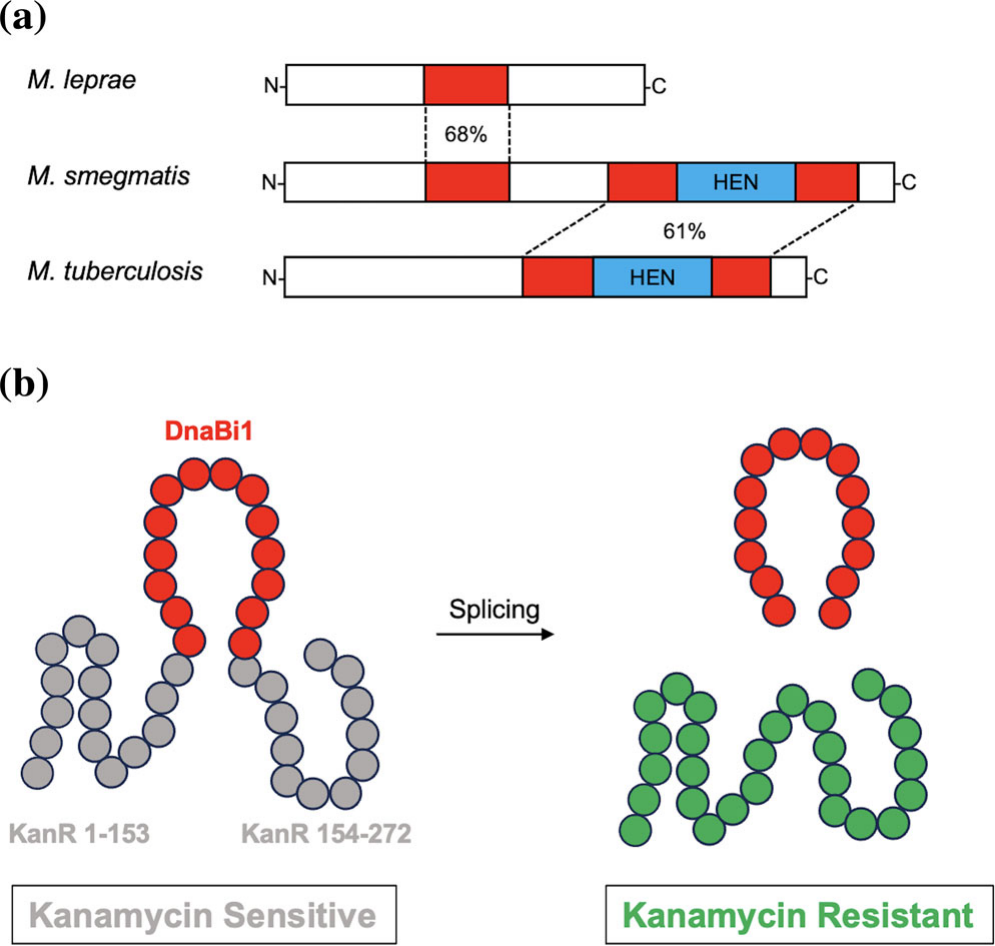
DnaB *in*tervening pro*teins* (inteins) in *Mycobacterium leprae*, *Mycobacterium smegmatis*, and *Mycobacterium tuberculosis*, and the kanamycin intein splicing reporter (KISR). (a) Inteins within DnaB helicase genes of some mycobacteria. Line diagrams of *M. leprae*, *M. smegmatis*, and *M. tuberculosis* DnaB inteins. Exteins, white; inteins, red; homing endonuclease, blue. Length to approximate scale. Percentage indicates sequence identity shared between inteins. (b) Cartoon of KISR. *M. smegmatis* DnaBi1 is inserted between residues 153 and 154 of KanR. When the intein splices out, a functioning KanR protein that confers resistance to the antibiotic kanamycin is formed. Each ball represents approximately 10 residues.

Nearly half of the genomes within the *Actinomycetota* (formerly *Actinobacteria*) phylum contain inteins (Novikova et al., 2014, 2016). This phylum has several bacteria that act as important human pathogens, including those within the *Mycobacterium* genus. DnaB inteins have been identified in *Mycobacterium tuberculosis*, *Mycobacterium leprae*, and other nontuberculous mycobacterial pathogens, and two inteins reside within the ATPase domain of DnaB from the nonpathogenic model organism *Mycobacterium smegmatis* (Wall et al., 2021). (Figure 1a). The first intein, DnaBi1, is a mini‐intein, as it is comprised of a single domain that catalyzes its splicing out of DnaB (Mills et al., 2014; Wood et al., 2023). The second intein, DnaBi2, contains a nested homing endonuclease domain that might contribute to DnaBi2 mobility and evolutionary conservation within the *M. smegmatis* DnaB helicase (Gogarten & Hilario, 2006). Inteins homologous to *M. smegmatis* DnaBi1 and DnaBi2 are found within *M. leprae* (DnaBi1) and *M. tuberculosis* (DnaBi2) (Figure 1a).

*M. smegmatis* DNA replication and survival almost certainly depend on splicing of both DnaB inteins, as DnaBi1 is located in the ATP‐binding P‐loop and DnaBi2 is found within a DNA‐binding loop (Kelley et al., 2018). However, it is currently unclear whether such events are regulated in vivo. Previously, it was found that, while *M. smegmatis* DnaBi2 splices rapidly when expressed in *Escherichia coli*, *M. smegmatis* DnaBi1 splicing is slow relative to the generation time of *M. smegmatis* (Kelley et al., 2018). As a means to better understand the factors that affect *M. smegmatis* DnaBi1 splicing in the cellular environment, a *k*anamycin *i*ntein *s*plicing *r*eporter (KISR), linking protein splicing to antibiotic resistance was constructed (Kelley et al., 2018; Lennon et al., 2021; Woods et al., 2020). In this artificial extein reporter, an aminoglycoside phosphotransferase, KanR (NCBI reference sequence WP_000018329.1), is interrupted by *M. smegmatis* DnaBi1 between residues 153 and 154, with Thr154 serving as the “+1” nucleophile required for splicing (Mills et al., 2014; Wood et al., 2023). During *M. smegmatis* DnaBi1 splicing, the KanR segments are rejoined, conferring kanamycin resistance to the cell (Figure 1b). Accordingly, if the intein is splicing‐active, then the host is resistant to kanamycin. If the intein is inactivated, either through mutation or by splicing inhibition, the host is sensitive to kanamycin. Using this reporter, we found that a variety of factors (e.g., reactive oxygen, reactive chlorine, and zinc) inhibit *M. smegmatis* DnaBi1 splicing (Kelley et al., 2018; Lennon et al., 2021; Woods et al., 2020). At the same time, factors that promote protein splicing in the context of this artificial extein reporter have not been identified.

In this work, we found that *M. smegmatis* DnaBi1 splicing is temperature sensitive. Indeed, intein splicing was inhibited at high, yet physiological, temperatures within the native mycobacterial host environment and within *E. coli* using our artificial extein KISR reporter. These results were substantiated in vitro using another non‐native extein splicing reporter. In a previous study, we found that overexpression of the chaperone GroEL increased protein stability of a splicing‐dependent biosensor (Son et al., 2024). Here, we demonstrate that GroEL overexpression results in at least a 100,000‐fold increase in survival in *E. coli* using our DnaBi1‐splicing‐dependent KISR system. We further show that the effect of GroEL overexpression on KISR‐dependent survival is greater under temperature‐induced protein folding stress. Mechanistically, we demonstrate that GroEL does not directly promote DnaBi1 splicing, but rather increases levels the unspliced precursor, which ultimately permits DnaBi1 more opportunities to splice. Taken together, our work details the first example of a protein chaperone affecting the splicing of an intein‐containing protein, suggesting that the precursor state for some intein‐containing proteins may be unstable compared to the mature spliced form. These findings not only have implications for the control and efficiency of various intein‐based technologies in the cellular environment, but they also raise compelling questions about how chaperones may influence protein splicing reactions in nature.

## 2 RESULTS

### 2.1 KanR‐DnaBi1 activity is inhibited by heat stress in *M. smegmatis*

**FIGURE 2:**
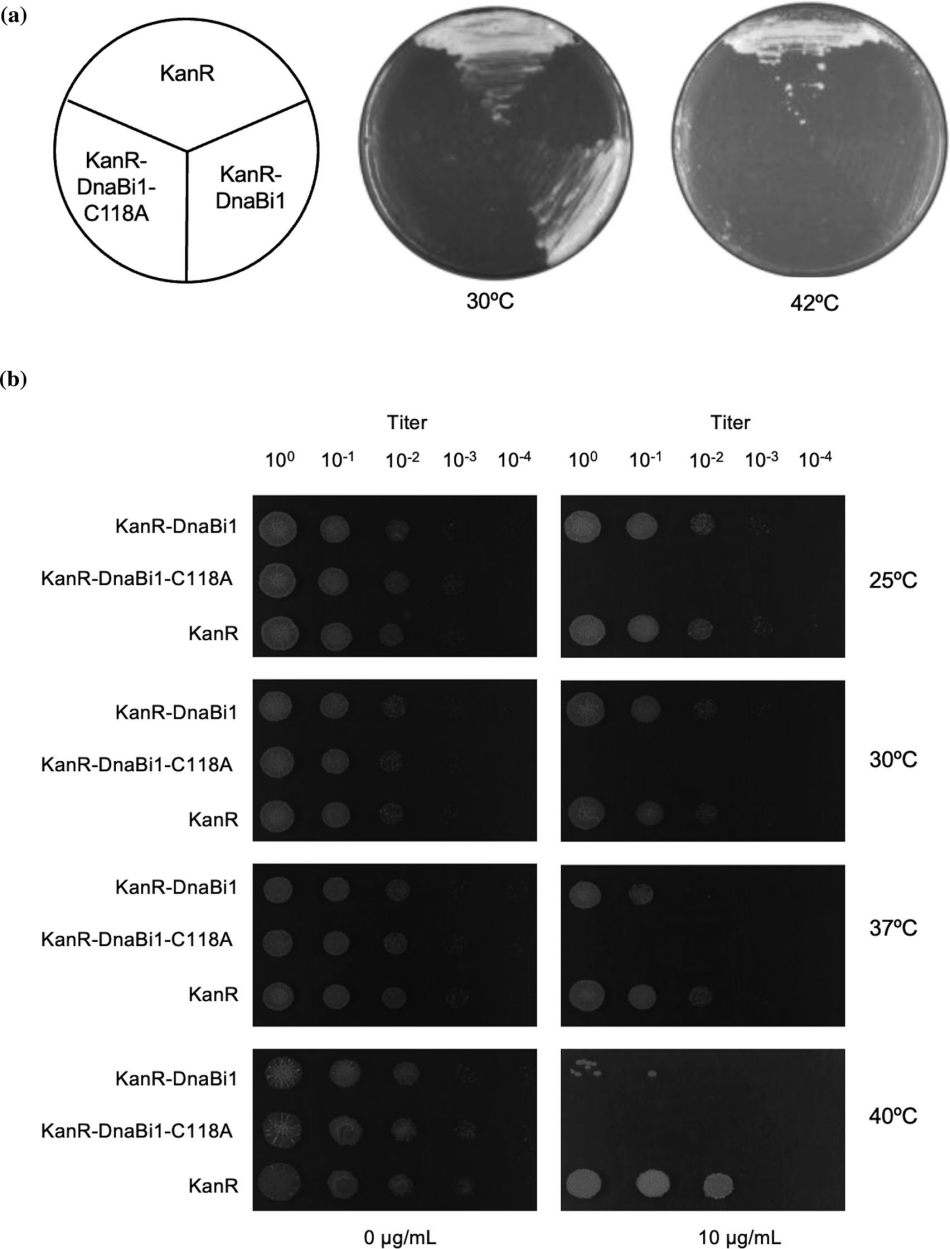
Elevated temperature inhibits DnaBi1 splicing in *Mycobacterium smegmatis*. (a) Growth of KanR‐DnaBi1, KanR‐DnaBi1‐C118A, and uninterrupted KanR in the presence of kanamycin at 30 and 42°C. (b) Spot titers of strains in panel (a) grown with either 0 or 10 μg/mL kanamycin at various temperatures indicated on the right.

As there is a paucity of studies that have examined protein splicing within the native cellular environment of the intein, we sought to identify stress factors that affect DnaBi1 splicing in *M. smegmatis*. To achieve this, *M. smegmatis* cells were transformed with a chromosome‐integrating plasmid that expressed the KanR‐DnaBi1 protein. Two additional strains—one expressing a splicing‐defective mutant (KanR‐DnaBi1‐C118A) and one expressing an intein‐lacking KanR—were used as negative and positive controls, respectively (Figure 2a). Of the limited number of conditions screened (not shown), incubation at elevated temperatures (42°C) resulted in a dramatic phenotype (Figure 2a). As expected, the KanR‐DnaBi1 expressing strain, but not the strain that expressed the splicing‐defective KanR‐DnaBi1‐C118A, grew well at 30°C. However, upon incubation at 42°C, the KanR‐DnaBi1 expressing strain presented a phenotype similar to that of the strain expressing the splicing‐defective KanR‐DnaBi1‐C118A (Figure 2a). In contrast, the strain expressing a KanR lacking an intein grew on kanamycin‐containing plates at 42°C. These results suggest that *M. smegmatis* DnaBi1 splicing is temperature sensitive within the artificial extein KISR context.

To better assess the effect of temperature on DnaBi1 splicing, we performed spot titer assays of each strain and observed the effect of different temperatures on kanamycin resistance. We found that while at 25 and 30°C, the KanR‐DnaBi1 expressing strain grew as well as the KanR expressing strain, the cells presented a clear reduced kanamycin resistance at 37°C (Figure 2b). The effect of temperature on the KanR‐DnaBi1 expressing strain was much greater at 40°C, as hardly any growth of this strain was recorded at this temperature on kanamycin‐containing plates. In contrast, robust growth of the KanR expressing strain was observed under the same conditions (Figure 2b). At all temperatures, the KanR‐DnaBi1‐C118A expressing strain presented no growth in the presence of kanamycin, and all strains grew equally well in the absence of kanamycin (Figure 2b). These findings suggest that, within the artificial extein KISR context, elevated temperatures inhibit *M. smegmatis* DnaBi1 splicing within the natural cellular environment of the intein.

### 2.2 *M. smegmatis* DnaBi1 splicing is inhibited by elevated temperatures in vitro

**FIGURE 3:**
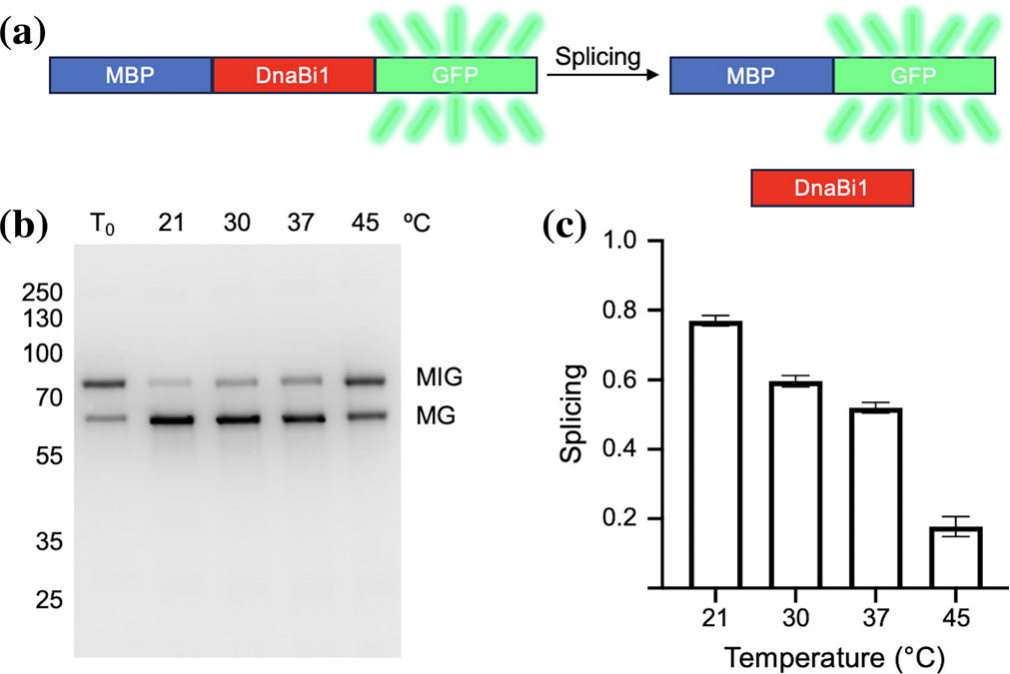
*Mycobacterium smegmatis* DnaB1 *in*tervening pro*teins* (intein) splicing is inhibited by increasing temperatures. (a) Diagram of MBP‐Intein‐GFP (MIG) reporter with the maltose‐binding protein (MBP; blue) as the N‐extein, followed by the *M. smegmatis* DnaB1 intein (DnaBi1; red), followed by the superfolder green fluorescent protein (GFP; green) as the C‐extein. GFP‐containing products MIG (precursor) and MG (ligated exteins) are visualized by in‐gel fluorescence. (b) Splicing of *M. smegmatis* DnaB1 intein following incubation at various temperatures for ~20 h. Time zero (*T*_0_) represents the level of products following expression. The gel is representative from at least three independent experiments. To the left of the gel, molecular weights (kDa) are shown. To the right of the gel, GFP‐containing products MIG and MG are indicated. (c) Quantification of splicing (see methods) from panel (b). The fraction spliced is the average from three independent experiments, and the error bars indicate the standard deviation of those three experiments.

To examine splicing inhibition by temperature directly, we measured *M. smegmatis* DnaBi1 splicing in vitro using a previously established splicing reporter where the maltose‐binding protein (MBP) is the N‐extein and superfolder green fluorescent protein (GFP) is the C‐extein (MBP‐Intein‐GFP or precursor (MIG); Figure 3a). The *M. smegmatis* DnaBi1 intein is flanked by 10 residues from the natural amino acid context. Thus, following MIG expression in *E. coli*, splicing in cell lysates can be assayed, and GFP‐containing products, including the unspliced precursor, ligated exteins, and off‐pathway cleavage products, can be visualized in‐gel using semi‐native sodium dodecyl sulfate–polyacrylamide gel electrophoresis (SDS‐PAGE) (Weinberger & Lennon, 2021). We found that most *M. smegmatis* DnaBi1 is unspliced after expression, as previously observed (Time 0; Figure 3b) (Reitter et al., 2016; Topilina, Green, et al., 2015; Topilina, Novikova, et al., 2015). Following a 20‐h incubation, which is necessary due to the slow *M. smegmatis* DnaBi1 splicing kinetics previously measured in the MIG reporter (Kelley et al., 2018; Lennon et al., 2021; Woods et al., 2020), splicing in lysates can, nevertheless, be detected (Figure 3b). At temperatures ranging from 21 to 45°C, we observe a gradual reduction in splicing efficiency (Figure 3b,c). These findings agree with the KanR‐DnaBi1‐dependent survival results in *M. smegmatis*, confirming that *M. smegmatis* DnaBi1 activity is sensitive to increased temperatures.

### 2.3 GroEL overexpression increases KanR‐DnaBi1 splicing‐dependent survival

**FIGURE 4:**
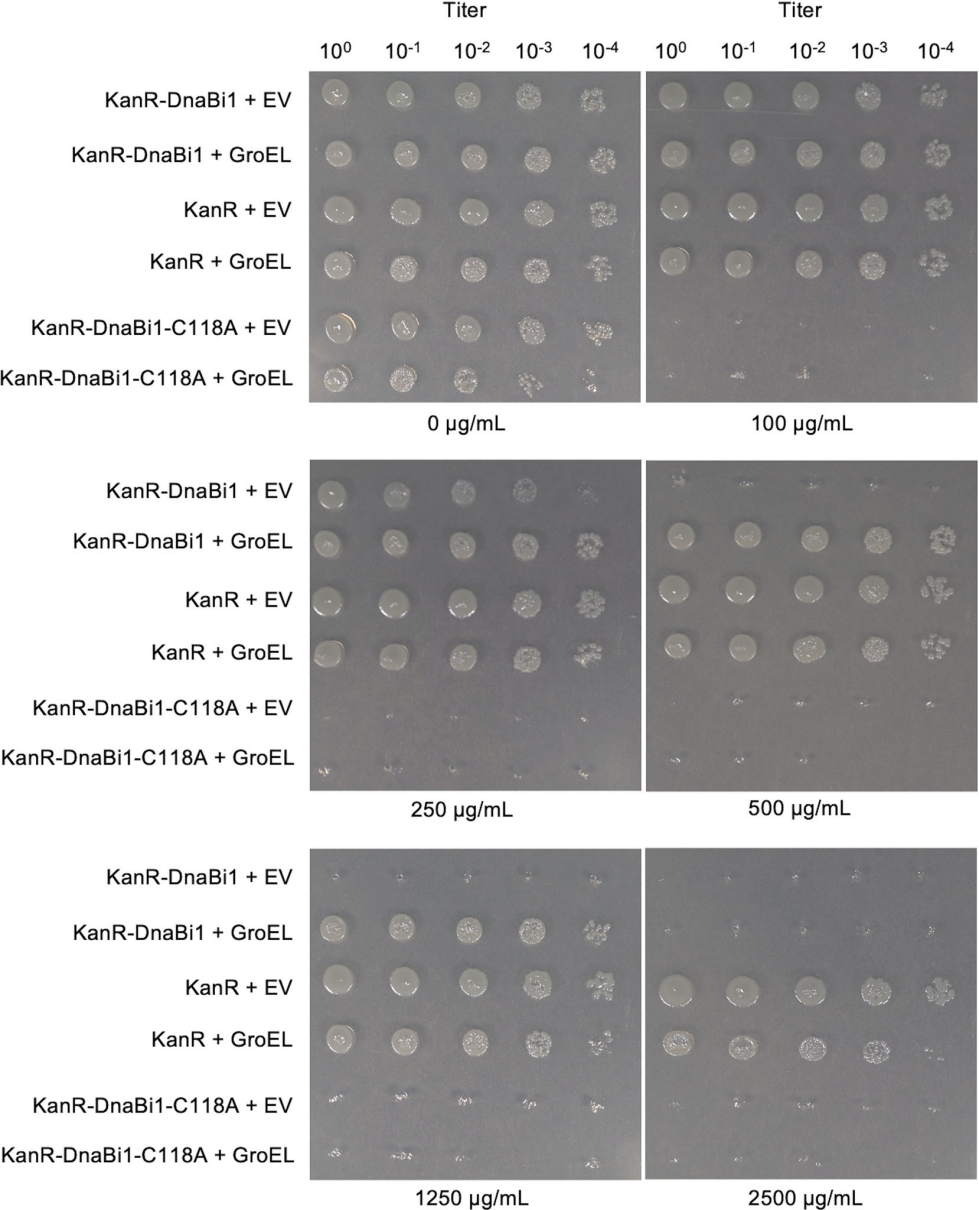
GroEL increases KanR‐DnaBi1‐dependent resistance to kanamycin. Spot titers at varying levels of kanamycin of strains described in text. L‐arabinose is present in all plates to induce GroEL expression. EV indicates empty vector control. Kanamycin concentrations are listed below each plate. Each plate is grown overnight at 39°C. The C118A mutation inactivates *Mycobacterium smegmatis* DnaBi1 splicing.

Using the *Pyrococcus horikoshii* RadA intein, we previously developed a splicing‐dependent biosensor to monitor protein stability in vivo, which was stabilized by GroEL in subsequent investigation (Son et al., 2024). In this previous work we did not determine whether GroEL, either directly or indirectly, promoted protein splicing, although results suggested that this could be a possibility. Given our findings regarding the temperature sensitivity of *M. smegmatis* DnaBi1 splicing, we hypothesized that overexpression of GroEL could increase *M. smegmatis* DnaBi1 splicing within the artificial extein KISR context and, in turn, allow for greater survival in the presence of increasing concentrations of kanamycin. To test this, two plasmids were transformed into *E. coli*. One plasmid constitutively expressed KanR‐DnaBi1 and another with *groEL* under the control of an arabinose‐inducible promoter. As controls, KanR without an intein, splicing‐inactive KanR‐DnaBi1‐C118A, and an empty vector backbone lacking GroEL were used. Initially, we performed quantitative spot titer assays for strains expressing KanR with and without GroEL, KanR‐DnaBi1 with and without GroEL, and a KanR‐DnaBi1‐C118A with and without GroEL (Figure 4). Titers of each strain were spotted on plates containing arabinose and a range of kanamycin concentrations. Plates were then grown at 39°C to impose a moderate folding stress. We observed that, while GroEL overexpression had no effect on KanR‐DnaBi1‐dependent survival at low kanamycin concentrations (0 and 100 μg/mL; Figure 4), it increased survival by at least 100,000‐fold at higher kanamycin concentrations (250–1250 μg/mL; Figure 4). In contrast, GroEL overexpression did not improve either KanR‐ or KanR‐DnaBi1‐C118A‐dependent survival. These results suggested that GroEL increased DnaBi1 protein splicing, either directly or indirectly, within the artificial extein KISR context. GroEL overexpression slightly reduced KanR‐dependent survival at the highest concentration of kanamycin tested (2500 μg/mL). This result, while seemingly counterintuitive, could potentially be explained by a reduction of KanR availability for kanamycin inactivation, as a result of GroEL‐KanR binding events. Similarly, the increased metabolic burden of GroEL overexpression may contribute to the reduced survival at 2500 μg/mL kanamycin. Note that higher kanamycin concentrations are used in *E. coli* titers because KanR, KanR‐DnaBi1, and KanR‐DnaBi1‐C118A are expressed from a high‐copy plasmid, whereas in *M. smegmatis* they are expressed from a single‐copy integrating plasmid.

To confirm that the GroEL overexpression was directly responsible for the improved KanR‐DnaBi1‐dependent survival, additional spot titers were performed for strains expressing KanR‐DnaBi1 and either the GroEL‐encoding plasmid or the empty vector. This time, plates with and without L‐arabinose and variable kanamycin concentrations were used (Figure S1). Following growth at 39°C for 20 h, both strains exhibited identical survival without GroEL induced expression in the absence of L‐arabinose. In contrast, L‐arabinose dramatically improved the survival of the strain that carried the *groEL*‐encoding plasmid at elevated kanamycin concentrations (Figure 4). This demonstrated that the induction of GroEL overexpression was sufficient for the increased KanR‐DnaBi1‐dependent survival compared to the empty vector control. We also observed that L‐arabinose improved the survival of the strain with KanR‐DnaBi1 and the empty vector even in the absence of kanamycin, presumably owing to the use of L‐arabinose as an additional carbon source.

**FIGURE 5:**
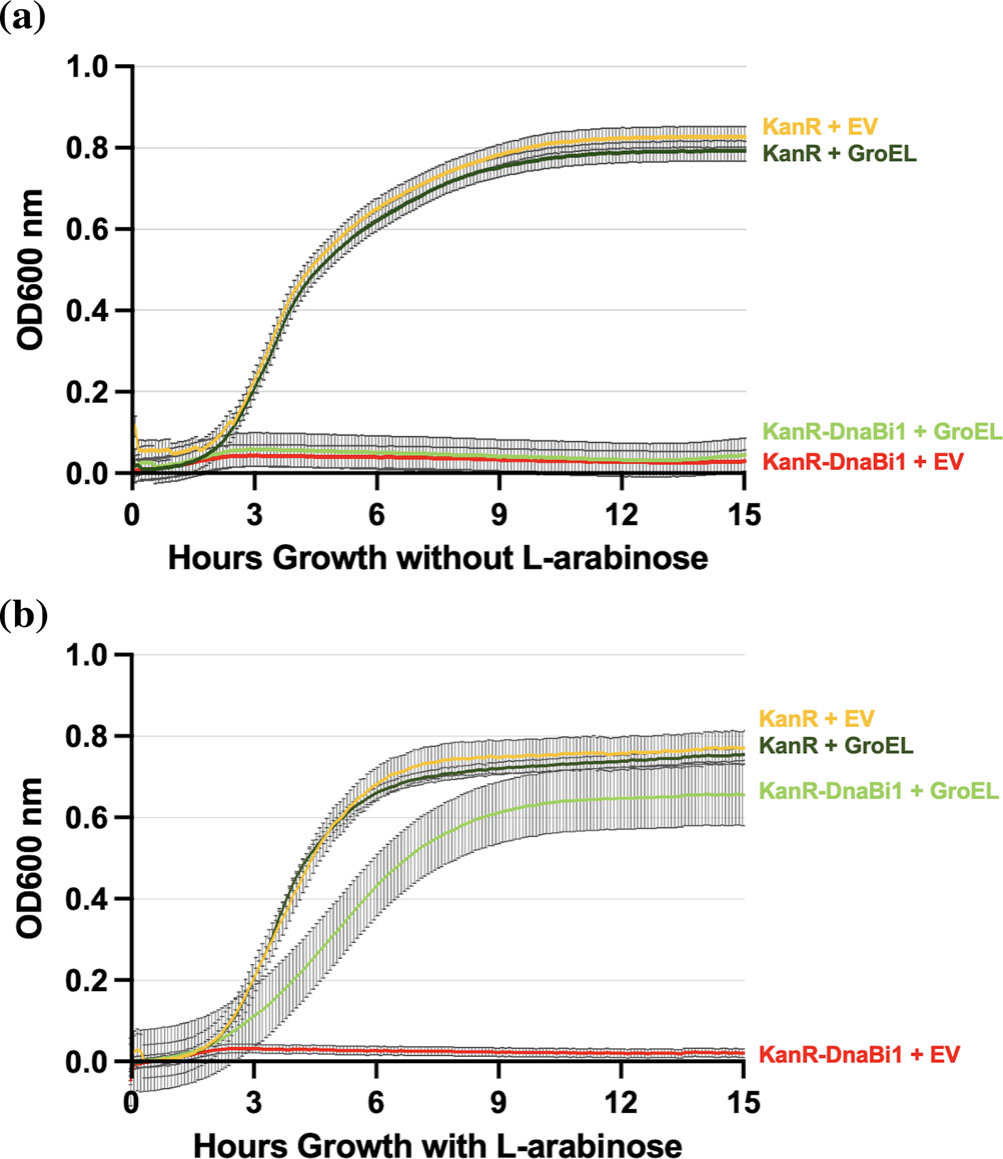
GroEL induction is required for increased KanR‐DnaBi1‐dependent resistance to kanamycin in liquid culture. Growth of strains described in the text in liquid culture (a) without or (b) with L‐arabinose at 500 μg/mL kanamycin. L‐arabinose induces GroEL expression. EV indicates empty vector control. Cultures were grown in a 96‐well plate at 37°C with shaking. Optical density averages are from three independent experiments, each containing three independent cultures. Error bars represent the standard deviation of all data at a given time point for each strain.

Following our observations of a relationship between GroEL overexpression and KanR‐DnaBi1 survival on solid media, we performed growth assays to examine this relationship in liquid culture using 96‐well plates. When designing these assays, we first determined the maximum kanamycin concentration that can be withstood by strains expressing KanR before displaying delayed or reduced growth. From these initial plates, we found that the KanR‐expressing strains can tolerate kanamycin concentrations up to 500 μg/mL without any detriment to their growth. However, both the KanR‐DnaBi1 and KanR‐DnaBi1‐C118A expressing strains were unable to survive a kanamycin concentration of 500 μg/mL (Figure 5a). Altogether, these results led us to use a kanamycin concentration of 500 μg/mL when examining whether GroEL overexpression would increase DnaBi1 splicing activity in liquid culture. Following incubation with shaking at 37°C, we observed that the KanR‐DnaBi1 strain with GroEL was only able to survive when grown in media containing L‐arabinose, whereas the KanR‐DnaBi1 strain harboring the empty vector failed to grow regardless of L‐arabinose concentration (Figure 5a,b). Conversely, the KanR‐expressing strains displayed similar growth when cultured with or without L‐arabinose, and regardless of GroEL expression (Figure 5). Altogether, our examination in liquid culture yielded results that reinforce what we observed from prior experiments on solid media, namely that GroEL expression improves KanR‐DnaBi1‐based resistance.

### 2.4 The effect of GroEL on KanR‐DnaBi1 splicing‐dependent survival is increased under folding stress

**FIGURE 6:**
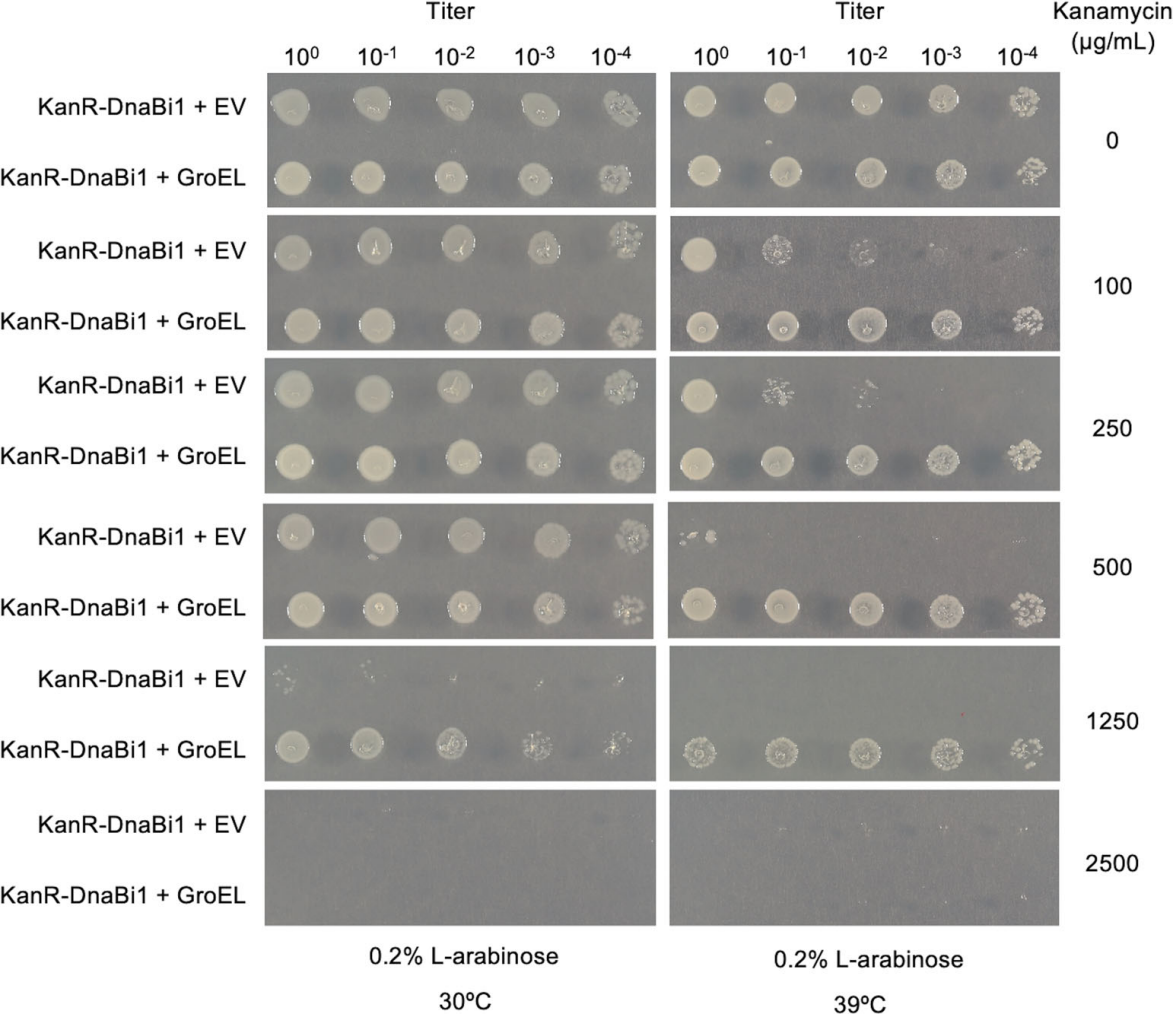
GroEL has a greater influence on KanR‐DnaBi1‐dependent resistance to kanamycin at higher temperature. Spot titer assay of strains described in the text at varying concentrations of kanamycin under non‐stressed (left; 30°C) versus stressed (right; 39°C) conditions. EV indicates empty vector control. Kanamycin concentrations are listed to the right of each row. Plates to the left are grown overnight at 30°C, and those to the right are grown overnight at 39°C.

**FIGURE 7:**
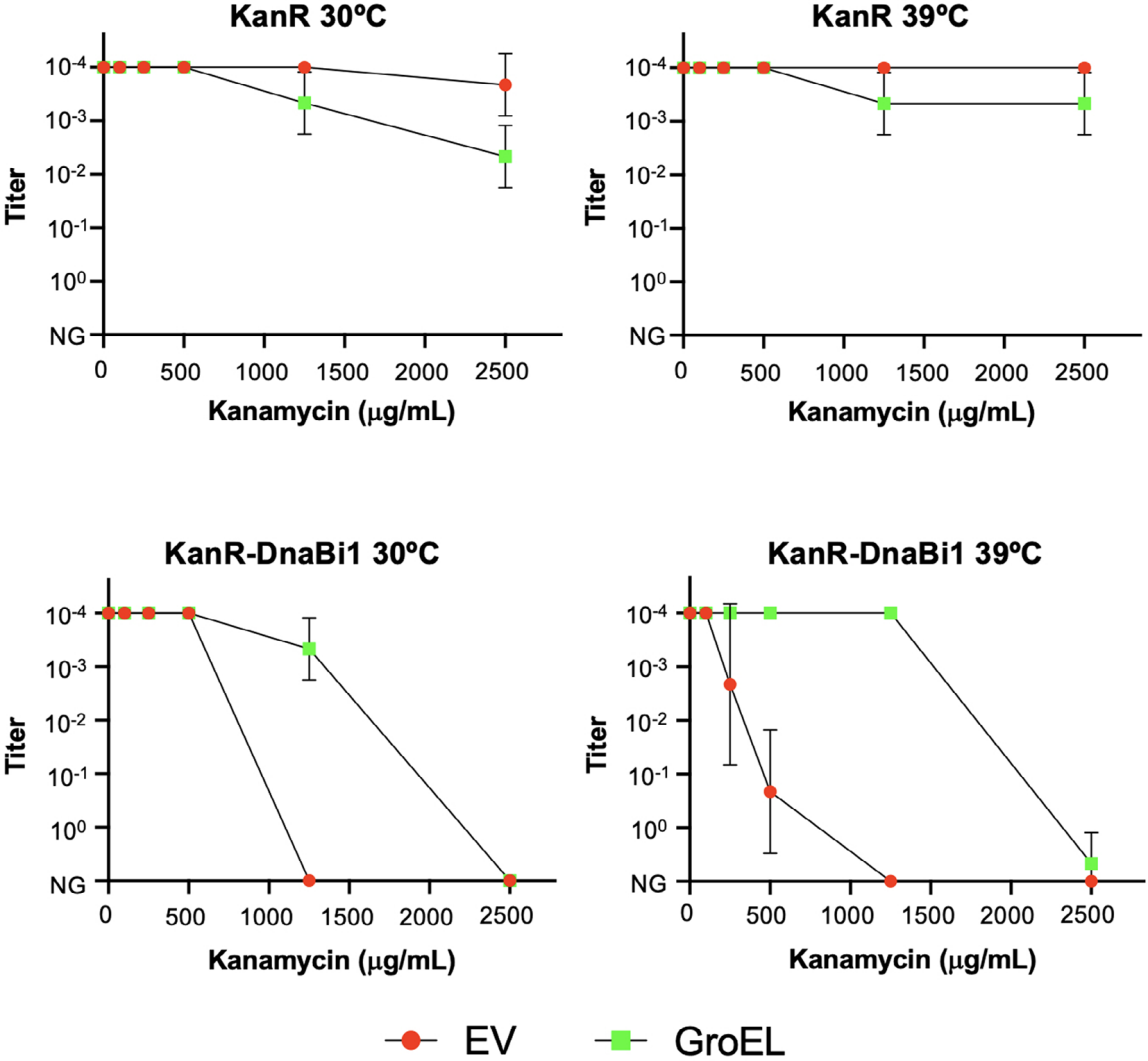
GroEL overexpression promotes KanR‐DnaBi1 splicing‐dependent survival. Survival curves of strains generated from spot titer assays from three biological replicates. KanR‐containing strains are in the top row and KanR‐DnaBi1‐containing strains are in the bottom row. Plates grown at 30°C are on the left and plates grown at 39°C are on the right. Empty vector control (EV)‐ and GroEL‐containing strains are denoted by the red circles and green squares, respectively. Quantification of titers is as follows, growth at 10^−4^ = 5, 10^−3^ = 4, 10^−2^ = 3, 10^−1^ = 2, 10^0^ = 1. No growth (NG) is scored as 0. If any growth occurs at a given titer, it is scored as the corresponding value. Each point is the average of the three biological replicates and error bars represent the standard deviation.

We next sought to examine how temperature influenced the effect of GroEL overexpression on KanR‐DnaBi1‐dependent survival. In the previous experiments (Figures 4 and S1), strains were grown at 39°C to cause protein folding stress. Since we also wanted to examine the non‐stress condition of 30°C, strains were spotted on two sets of plates with L‐arabinose and variable kanamycin concentrations and then grown at 30°C or 39°C (Figure 6). For strains that carried the empty vector, KanR‐DnaBi1‐dependent survival was decreased at 39°C compared to 30°C (Figure 6), in accordance with DnaBi1 splicing inhibition that we observed in *M. smegmatis* (Figure 2a,b). While GroEL overexpression increased KanR‐DnaBi1‐dependent survival at both 30 and 39°C, the degree to which GroEL promoted survival was much greater at 39°C, further reinforcing the relationship between GroEL and KanR‐DnaBi1‐dependent survival (Figure 7). Interestingly, opposite temperature‐ and GroEL‐dependent survival trends are observed for KanR alone compared to the KanR‐DnaBi1 fusion (Figure 7).

### 2.5 GroEL increases the stability of unspliced KanR‐DnaBi1

**FIGURE 8:**
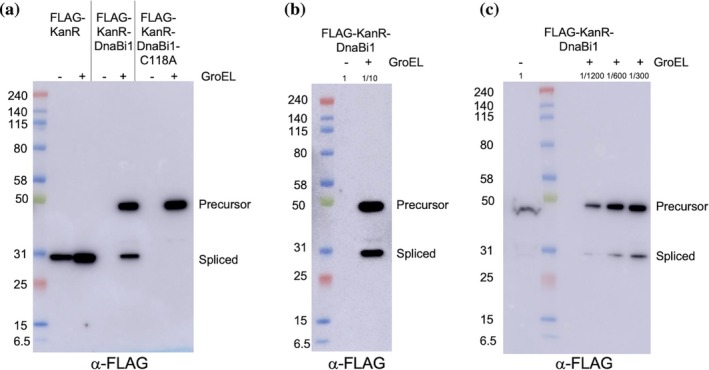
GroEL overexpression drastically stabilizes unspliced precursor. (a) Comparison of cellular lysates of FLAG‐KanR‐, FLAG‐KanR‐DnaBi1‐, and FLAG‐KanR‐DnaBi1‐C118A‐containing cells with either the empty vector (−GroEL) or GroEL overexpression (+GroEL) by western blot. (b) Comparison of different concentrations of cellular lysate of FLAG‐KanR‐DnaBi1‐containing cells with and without GroEL overexpression by western blot. In this panel, 10‐fold more lysate from the −GroEL cells is added compared to the +GroEL cells. (c) Comparison of different concentrations of cellular lysate of FLAG‐KanR‐DnaBi1‐containing cells as in panel B, but with 300‐, 600‐, and 1200‐fold more −GroEL cellular lysate by western blot. In all cases, cells were grown at 39°C in the presence of arabinose and absence of kanamycin. To the left of the gel, molecular weights (kDa) are shown.

To better understand the mechanism of increased KISR‐dependent survival in the presence of GroEL overexpression, we decided to measure the soluble levels of the KanR protein (spliced and unspliced) using western blotting. To achieve this, we fused a FLAG‐tag to the N‐terminus of each of our constructs (KanR, KanR‐DnaBi1, and KanR‐DnaBi1‐C118A). Initially, we confirmed via spot titer assay that the addition of the FLAG‐tag did not interfere with the kanamycin. Indeed, hardly any difference in kanamycin resistance of the tagged and untagged KISR versions could be observed (Figure S2). Next, we grew *E. coli* cultures harboring either FLAG‐KanR, FLAG‐KanR‐DnaBi1, or FLAG‐KanR‐DnaBi1‐C118A precursor, as well as a plasmid for GroEL overexpression or an empty vector control. Following growth at 39°C in the presence of L‐arabinose and in the absence of kanamycin (i.e., conditions where all strains, including those with KanR‐DnaBi1‐C118A, grew), an equal number of cells were harvested from plates and soluble proteins were probed by western blotting. As expected for FLAG‐KanR‐DnaBi1, a band corresponding to the spliced FLAG‐KanR was detected (31.9 kDa) (Figure 8a). A band corresponding to unspliced precursor (46.5 kDa) was also observed (Figure 8a). In contrast, only a band consistent in size with the unspliced precursor was detected for FLAG‐KanR‐DnaBi1‐C118A‐containing cells (Figure 8a). In the absence of GroEL overexpression, no bands consistent with either spliced or unspliced products were detectable for strains expressing either FLAG‐KanR‐DnaBi1 or FLAG‐KanR‐DnaBi1‐C118A (Figure 8a). We also probed the insoluble fraction, and we find that while the KanR‐DnaBi1 (wild type and C118A) fusion proteins are absent from the soluble fraction without GroEL, they are present in the insoluble fraction (Figure S3). Therefore, it is clear that GroEL supports increased soluble FLAG‐KanR‐DnaBi1 and FLAG‐KanR‐DnaBi1‐C118A protein levels.

For FLAG‐KanR, we observed a single band at the expected size both in the absence and in the presence of GroEL overexpression, with a stronger signal upon GroEL overexpression (Figure 8a). We estimate that GroEL overexpression led to approximately a four‐fold increase in soluble FLAG‐KanR levels (Figure S4). To assess the degree to which GroEL overexpression increases the soluble levels of spliced and unspliced products for strains expressing FLAG‐KanR‐DnaBi1, we loaded different concentrations of soluble cellular proteins for strains with the empty vector compared to those overexpressing GroEL. When 10‐fold less soluble protein for cells overexpressing GroEL was loaded, we again observed no products in cells with the empty vector (Figure 8b). Upon further dilution, we found that approximately 1200‐fold fewer cells from GroEL overexpressing strains are sufficient to give signal comparable to the signal detected in cells with the empty vector (Figure 8c). In three independent experiments, using this dilution‐based approach, we observed a 1533‐fold increase (standard deviation 215) in soluble FLAG‐KanR‐DnaBi1 precursor with GroEL overexpression. Conversely, we only observe a 2.5‐fold increase (standard deviation 0.6) in soluble levels of FLAG‐KanR. Overall, our western blots indicated that, while soluble FLAG‐KanR levels are increased two‐ or three‐fold upon GroEL overexpression, soluble levels of FLAG‐KanR‐DnaBi1 and FLAG‐KanR‐DnaBi1‐C118A increased in excess of 1000‐fold, indicating that the effect of GroEL on protein levels was much greater for the intein‐containing reporter than for KanR.

## 3 DISCUSSION

Identifying novel factors that can influence intein activity is critical to our understanding of how protein splicing proceeds in the cellular environment. Previous work has shown that several solution conditions (e.g., temperature, salt, reactive oxygen species, etc.), as well as nucleic acid, can alter the rate and accuracy of protein splicing (Belfort, 2017; Callahan et al., 2011; Ciragan et al., 2016; Green et al., 2019; Kelley et al., 2018; Lennon et al., 2016, 2018, 2019, 2021; Lennon & Belfort, 2017; Liman et al., 2024; Mills & Paulus, 2001; Reitter et al., 2016; Schneider et al., 2024; Topilina, Green, et al., 2015; Topilina, Novikova, et al., 2015; Wood et al., 2023, 2020; Yalala et al., 2022). Using our KISR system (Figure 1), in this work we find that elevated temperature inhibits DnaBi1 splicing‐dependent survival in the native host of the intein, *M. smegmatis* (Figure 2). While our KISR system relies on artificial exteins, very few studies have examined splicing within the native host environment (Kelley et al., 2018; Liman et al., 2024; Schneider et al., 2024). Next, we demonstrate that temperature also has a large influence on the rate of *M. smegmatis* DnaBi1 splicing using our in vitro MIG reporter (Figure 3). We also show that overexpression of the chaperone GroEL increases *M. smegmatis* DnaBi1 splicing‐dependent survival in *E. coli* by at least 100,000‐fold, and the effect of GroEL is amplified under folding stress (Figures 4, 5, 6, 7 and S1). Finally, we find that GroEL drastically increases the stability of the unspliced KanR‐DnaBi1 precursor by greater than 1000‐fold, while only stabilizing the non‐intein‐containing KanR by around three‐fold (Figures 8 and S3).

Results within both of our artificial extein reporters, MIG and KISR, demonstrate that *M. smegmatis* DnaBi1 activity is lower higher temperature (Figures 3, 6, and 7). We hypothesize that the folding of this intein is compromised by elevated temperature, rather than direct effects on *M. smegmatis* DnaBi1 chemistry as previously observed (Kelley et al., 2018; Lennon et al., 2021; Woods et al., 2020). Our results suggest that GroEL does not directly lead to increased intein catalysis, but rather that GroEL stabilizes the unspliced precursor protein, which in turn results in a greater pool of precursor protein that can eventually splice independently (Figure 8). It is unclear, however, if GroEL overexpression influences the efficiency of precursor conversion to ligated exteins. In the case of our splicing‐dependent reporter, our results show that the intein‐containing precursor (KanR‐DnaBi1 and KanR‐DnaBi1‐C118A) is unstable compared to the mature ligated exteins (KanR), as no product is observed in the absence of GroEL overexpression (Figure 8a). While our results rely on an artificial protein splicing reporter, due to the enormous effect of GroEL on precursor stabilization and, in turn, indirectly the level of ligated exteins, we hypothesize that chaperones may promote splicing within the cellular context when the precursor is inherently unstable or when splicing must occur under conditions of protein folding stress. In agreement with this hypothesis, we observe that the magnitude of the effect of GroEL is substantially higher at 39°C compared to 30°C (Figures 6 and 7). Therefore, we further hypothesize that, by stabilizing the precursor, rather than promoting intein catalysis directly, GroEL ultimately allows more opportunities for productive protein splicing, which in turn leads to more mature KanR. As GroEL works together with GroES in vivo to promote cellular protein folding, endogenous GroES may play a role in helping to stabilize the KanR‐DnaBi1 precursor, although this was not tested. GroEL overexpression alone, however, has been shown to promote protein folding (Novikova et al., 2016; Son et al., 2024).

Furthermore, given the evolutionary conservation of GroEL across bacterial species, we suggest a functional link between the GroEL effect presented here on protein splicing in *E. coli* and the sensitivity of *M. smegmatis* DnaBi1 to elevated temperatures. It is likely that at elevated temperature DnaBi1 is found in competition for GroEL binding with many more misfolded proteins. Under these conditions, DnaBi1 splicing can be limited by the GroEL availability in a manner that contributes to the observed temperature sensitivity of DnaBi1 splicing in *M. smegmatis*.

Taken together, our results not only identify a physiologically relevant condition where *M. smegmatis* DnaBi1 splicing is inhibited in vitro and in vivo within our artificial extein reporters, but also demonstrate for the first time that protein splicing can be promoted by a chaperone by increasing the pool of unspliced precursor. This finding suggests that protein–protein interactions, rather than just solution conditions, may influence some splicing reactions in nature. As many intein‐containing proteins, such as those involved in DNA transactions, form interactions within larger complexes, our results raise the possibility that interaction‐mediated intein removal could be used as a regulatory strategy to control intein‐containing host protein activation. We hypothesize that DnaBi1 could potentially serve as a pause button to delay DNA replication under protein folding stress, but we readily concede that further experimentation within *M. smegmatis* and in the native extein context is required to establish this connection to our results. Overall, our demonstration that a chaperone can indirectly promote protein splicing through precursor stabilization not only increases our understanding of the factors that may influence protein splicing in vivo, but also informs strategies to enhance the efficiency of intein‐based protein engineering techniques.

## 4 MATERIALS AND METHODS

### 4.1 *E. coli* plasmids and strains

Plasmids (Table 1) were transformed or co‐transformed into BL21(DE3) (New England Biolabs), and this strain was used for all *E. coli experiments*.

**TABLE 1:** *Escherichia coli* plasmids.

Plasmid	Resistance	References
pACYC‐MIG‐DnaBi1	Chloramphenicol	Topilina, Novikova, et al. (2015)
pUC4‐KanR	Ampicillin	Topilina, Novikova, et al. (2015)
pUC4‐KanR‐DnaBi1	Ampicillin	Topilina, Novikova, et al. (2015)
pUC4‐KanR‐DnaBi1‐C118A	Ampicillin	Topilina, Novikova, et al. (2015)
pUC4‐FLAG‐KanR	Ampicillin	This study
pUC4‐FLAG‐KanR‐DnaBi1	Ampicillin	This study
pUC4‐FLAG‐KanR‐DnaBi1‐C118A	Ampicillin	This study
pBAD33‐Empty	Chloramphenicol	Son et al. (2024)
pBAD33‐GroEL	Chloramphenicol	Son et al. (2024)

### 4.2 MIG splicing assays

*E. coli* BL21(DE3) containing pACYC‐MIG‐DnaBi1 was inoculated in 5 mL lysogeny broth (LB) medium containing chloramphenicol (25 μg/mL) and incubated for 20 h at 37°C shaking at 250 RPM. The next day, 1 mL of each culture was diluted into 50 mL LB medium containing chloramphenicol (25 μg/mL) and incubated for 2 h at 37°C before adding 50 μL of 1M Isopropyl β‐D‐1‐thiogalactopyranoside to induce protein expression and incubated for another 3 h at 16°C, shaking at 250 RPM. Cultures were pelleted using a centrifuge and resuspended in 1 mL MIG buffer (50 mM Tris, 10% v/v glycerol) before being lysed via sonication. Insoluble elements were removed with centrifugation. Samples were aliquoted and incubated at various temperatures (21, 30, 37, and 45°C) for 20 h to allow protein splicing in vitro. One aliquot was prepared with Laemmli sample buffer (Bio‐Rad) to a concentration of 1× and then frozen (Time 0). After a 20‐h incubation, each aliquot was centrifuged to separate any aggregated proteins, then Laemmli sample buffer (Bio‐Rad) was added to each lysate to a final concentration of 1× (like the Time 0). The samples were not heated further to avoid GFP denaturation. Proteins were separated using 8%–16% Mini‐PROTEAN TGX gels (Bio‐Rad). After SDS‐PAGE, the gel was imaged with an Amersham Imager 680 (GE Healthcare) to detect fluorescent products. Densitometric quantification was used to determine the levels of fluorescent products using ImageJ (https://imagej.nih.gov/ij). Splicing was calculated as [(LE/LE + *P*)*t*_20_ − (LE/LE + *P*)*t*_0_]/[1 − (LE/LE + *P*)*t*_0_], where LE is ligated exteins, *P* is precursor, *t*_0_ is time zero, and *t*_20_ is after incubation for 20 h at the indicated temperature.

### 4.3 *M. smegmatis* spot titer assays

The *M. smegmatis* MC2155 strain containing the integrating plasmid pMV306 clones (KanR, KanR‐DnaBi1, and KanR‐DnaBi1‐C118A) were grown at 30°C in Middlebrook 7H9 broth containing 0.05% (v/v) Tween‐80 and 0.4% (v/v) glycerol. Cultures were diluted to a turbidity of OD_600_ = 0.3, and serially diluted 10‐, 100‐, 1000‐ and 10,000‐fold in 7H9 broth. Ten microliters of each dilution was spotted onto Middlebrook 7H10 agar plates supplemented with 0.4% glycerol and either 0 or 10 μg/mL kanamycin. Plates were incubated at 25, 30, 37 and 40°C.

### 4.4 *E. coli* spot titer assays

*E. coli* strains containing both pUC4K‐ and pBAD33‐based plasmids were inoculated into 5 mL of LB broth containing ampicillin (100 μg/mL) and chloramphenicol (25 μg/mL), then incubated at 30°C while shaking at 250 RPM for 20 h. The next day, cultures were diluted to 1 OD_600_ and serially diluted further in LB broth to OD_600_ readings of 10^0^, 10^−1^, 10^−2^, 10^−3^, and 10^−4^. About 1.25 μL of each dilution was spotted onto LB agar plates containing 100 μg/mL ampicillin, 25 μg/mL chloramphenicol, varying amounts of kanamycin (0–2500 μg/mL), and 0.2% L‐arabinose where applicable. Plates were incubated for 20 h at 30 or 39°C and photographed after 20 h of growth.

### 4.5 *E. coli* 96‐well plate growth assays

Four *E. coli* strains—containing pUC4‐KanR + pBAD33‐Empty, pUC4‐KanR + pBAD33‐GroEL, pUC4‐KanR‐DnaBi1 + pBAD33‐Empty, and pUC4‐KanR‐DnaBi1 + pBAD33‐GroEL respectively—were inoculated into 5 mL of LB broth containing ampicillin (100 μg/mL) and chloramphenicol (25 μg/mL), then incubated with shaking (250 RPM) at 30°C for 20 h. The following day, the cultures were diluted to an optical density at 600 nm (OD_600_) readings of 0.02 in 5 mL of LB broth containing the same concentration of ampicillin and chloramphenicol. In a well of a clear 96‐well plate, 100 μL of these dilutions were plated into 100 μL of LB broth with ampicillin (100 μg/mL), chloramphenicol (25 μg/mL), kanamycin (500 μg/μL), and 0.04% L‐arabinose where applicable. This resulted in a total liquid culture volume of 200 μL, with an inoculation OD_600_ of 0.01 into LB broth containing 100 μg/μL ampicillin, 25 μg/mL chloramphenicol, 500 μg/μL kanamycin, and, if called for, 0.02% L‐arabinose. Each strain was tested under the absence and presence of L‐arabinose, with three replicates per growth condition. An additional set of blank wells containing LB with 100 μg/mL ampicillin and 25 μg/mL chloramphenicol were present to provide a baseline. Once the experimental and blank cultures were plated, the 96‐well plate was covered with a clear lid to limit evaporation, but not sealed. The plates were then transferred to a SPECTROstar Nano, where they were incubated with double orbital shaking (300 RPM) at 37°C for 20 h (BMG LabTech). Absorbance was measured every 5 min using a 2 mm spiral well scan to track culture growth. Three growth assays were performed, resulting in nine replicates per each strain under a specific growth condition.

### 4.6 Western blotting

Strains containing a FLAG‐tag were grown on LB agar plates containing 100 μg/mL ampicillin, 25 μg/mL chloramphenicol, and 0.2% L‐arabinose; two sets of plates were incubated at 30 and 39°C for 20 h. Each plate was then flooded—LB broth was added, and cells were gently scraped off—to transfer cells to a liquid media. Each culture was then diluted to an OD_600_ of 10; 1 mL of these dilutions was pelleted with a centrifuge, resuspended in 1 mL MIG buffer (50 mM Tris, 10% v/v glycerol), and lysed via sonication. Insoluble elements were removed via centrifugation. When probing insoluble proteins, fractions were resuspended in 1 mL of 8M Urea. Seventy microliters of each lysate was added to 5 μL of 1M dithiothreitol and 25 μL 4× lithium dodecyl sulfate Sample Buffer (GenScript) for a final concentration of 1×. The samples were then heated at 95°C for 5 min. Proteins were then separated using an 8%–16% Bis‐Tris SurePAGE gel (GenScript). After SDS‐PAGE, the proteins were transferred to a polyvinylidene fluoride membrane using a fast wet transfer system (GenScript). The membrane was then placed in a blocking buffer with 5% w/v dry milk in 1× tris‐buffered saline with tween‐20 (TBS‐T) (50 mM Tris‐Cl, 150 mM NaCl, 1% v/v Tween 20) at 4°C for 20 h. After 20 h, the horseradish peroxidase‐conjugated FLAG‐antibody (GenScript) (0.5 μg/mL) was added to the buffer and allowed to rock for 1 h. The membrane was then removed from the antibody and washed with 1× TBS‐T buffer three times. Super Signal West Pico Plus (Thermo Scientific) was used to induce chemiluminescence on the membrane, which was detected by an Amersham Imager 680 (GE Healthcare). Densitometric quantification was used to determine the levels of each product using ImageJ.

## AUTHOR CONTRIBUTIONS

**Christopher W. Lennon:** Supervision; conceptualization; investigation; funding acquisition; writing – original draft; writing – review and editing; methodology. **Eyal Gur:** Conceptualization; investigation; funding acquisition; writing – original draft; writing – review and editing; supervision; methodology. **John S. Smetana:** Investigation; data curation; writing – review and editing; methodology; writing – original draft; validation. **Tia M. Ariagno:** Investigation; writing – review and editing; data curation; methodology. **Christopher R. Powell:** Investigation; writing – original draft; methodology; validation; writing – review and editing; data curation. **Joel Weinberger II:** Investigation; writing – review and editing. **Noy Bagdadi:** Investigation; writing – review and editing; methodology; data curation. **Hazel Thomas:** Investigation; writing – review and editing.

## Supporting information


**Data S1.** Supporting Information.

## Data Availability

The data that support the findings of this study are openly available in Figshare at https://figshare.com, reference number 31967682.
