# Supplementary material for: A protein chaperone can stabilize the intein‐containing precursor to indirectly promote protein splicing
Source: Protein Sci. 2026 Aug 14;35(9):e70768. doi: 10.1002/pro.70768 (PMC13476343; doi:10.1002/pro.70768)
Supplement: Supplementary file 1 [file PRO-35-e70768-s001.pdf]

SUPPLEMENTARY MATERIALS

A protein chaperone can promote intein splicing through precursor stabilization

John S. Smetana[1#], Christopher R. Powell[1#], Noy Bagdadi[2#], Tia M. Ariagno[1], Hazel Thomas[1], Joel Weinberger II[1], Eyal Gur[2], Christopher W. Lennon[1*]

[1]Department of Biological Sciences, Murray State University, Murray, KY, United States

[2]Department of Life Sciences, Ben-Gurion University of the Negev, Beer-Sheva, Israel

[*]Correspondence: clennon1@murraystate.edu

[#]Authors contributed equally

SUPPLEMENTAL FIGURE 1

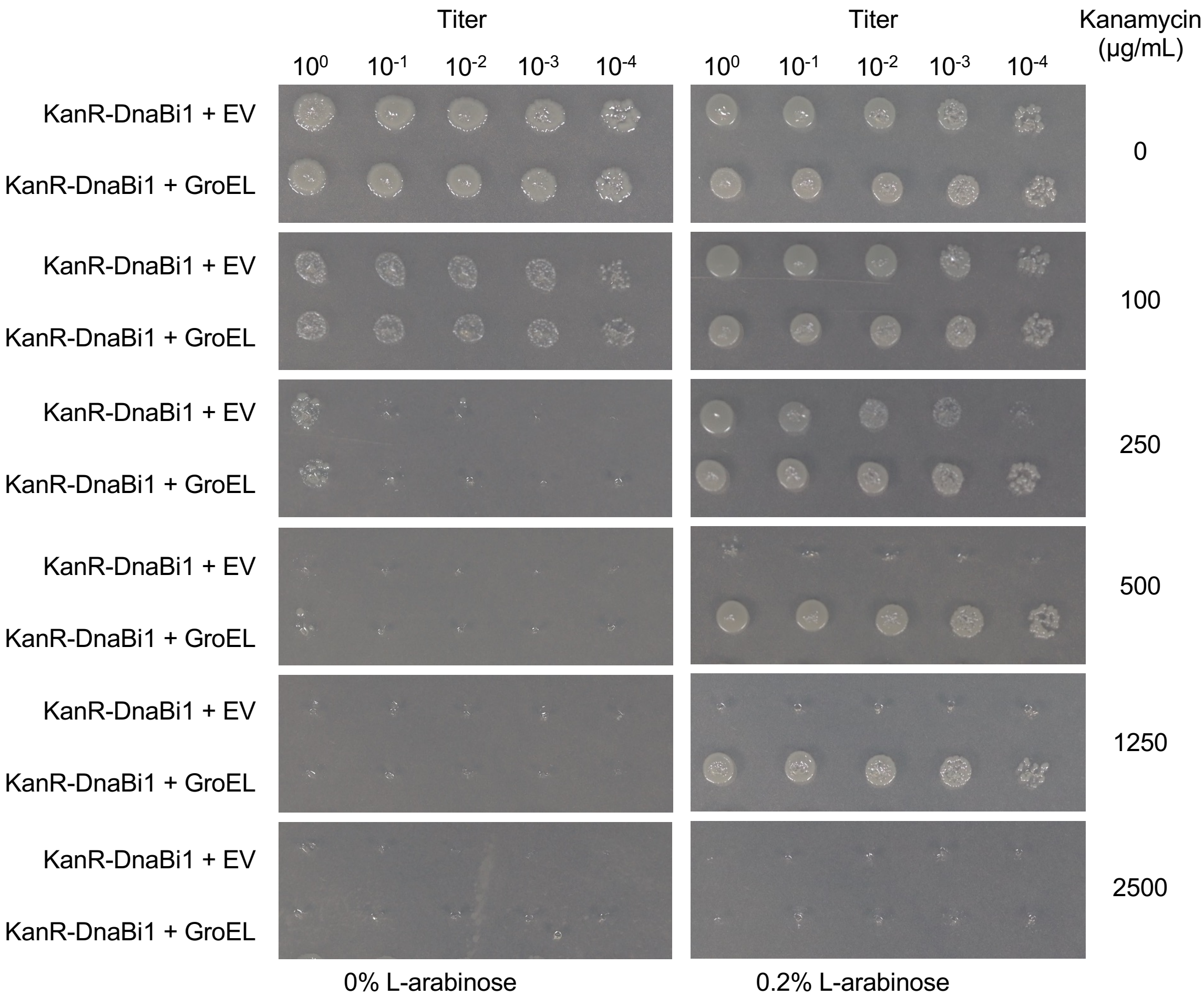

Supplemental Figure 1: GroEL induction is required for increased KanR-DnaBi1-dependent resistance to kanamycin compared to the empty vector control. Spot titer assay of strains described in the text with or without L-arabinose to at varying concentrations of kanamycin. L-arabinose, which induces GroEL expression, concentrations are below each column of plates. EV indicates empty vector control. Kanamycin concentrations are listed to the right of each row. Each plate is grown overnight at 39°C.

SUPPLEMENTAL FIGURE 2

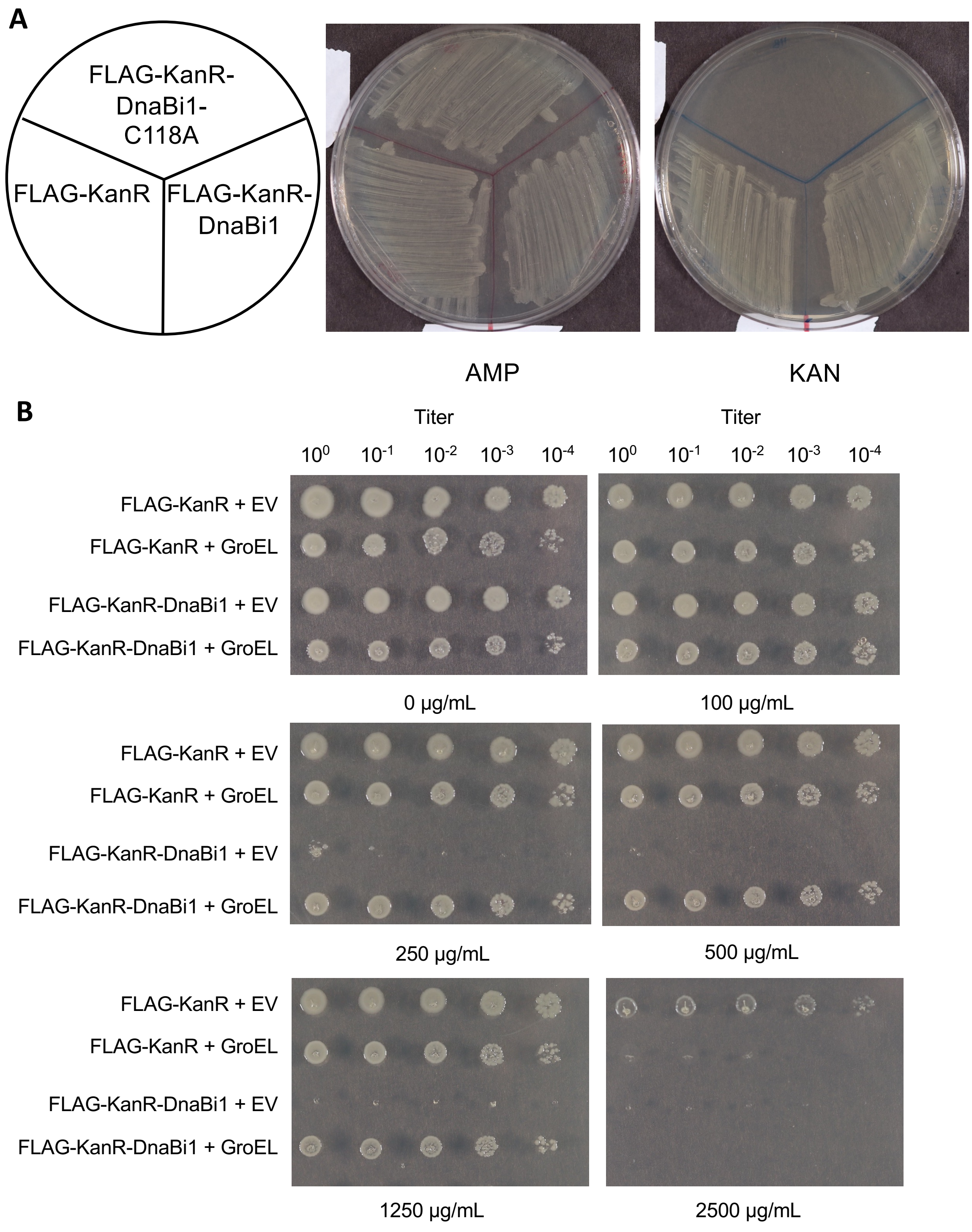

Supplemental Figure 2: FLAG-tag does not interfere with KanR- or KanR-DnaBi1-dependent resistance to kanamycin. (A) FLAG-KanR, FLAG-KanR-DnaBi1, and FLAG-KanR-DnaBi1-C118A provide resistance to ampicillin, but only FLAG-KanR and FLAG-KanR-DnaBi1 confer resistance to kanamycin. The C118A mutation blocks *M. smegmatis* DnaBi1 splicing. (B) Spot titer assay of strains of FLAG-tagged strains as performed in in Figure 2 at varying concentrations of kanamycin. EV indicates empty vector control. Kanamycin concentrations are listed to the right of each row. Plates were grown overnight at 39°C.

SUPPLEMENTAL FIGURE 3

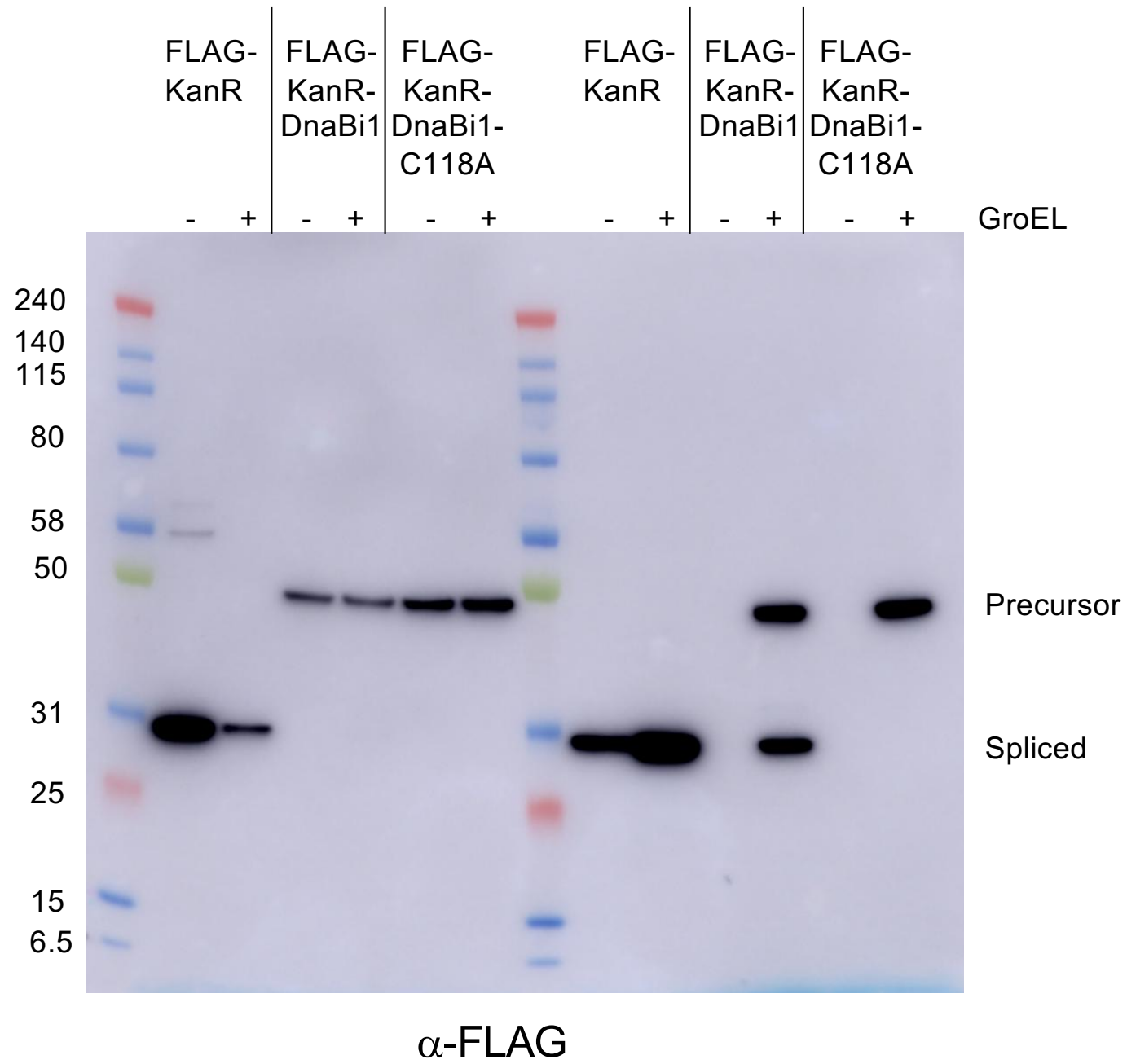

Supplemental Figure 3: GroEL overexpression increases FLAG-KanR and FLAG-KanR-DnaBi1 (WT and C118A) soluble protein levels. Comparison of insoluble and soluble protein levels of FLAG-KanR and FLAG-KanR-DnaBi1 (WT and C118A) with either the empty vector (-GroEL) or GroEL overexpression (+GroEL) by western blot. To the left of the gel, molecular weights (kDa) are shown.

SUPPLEMENTAL FIGURE 4

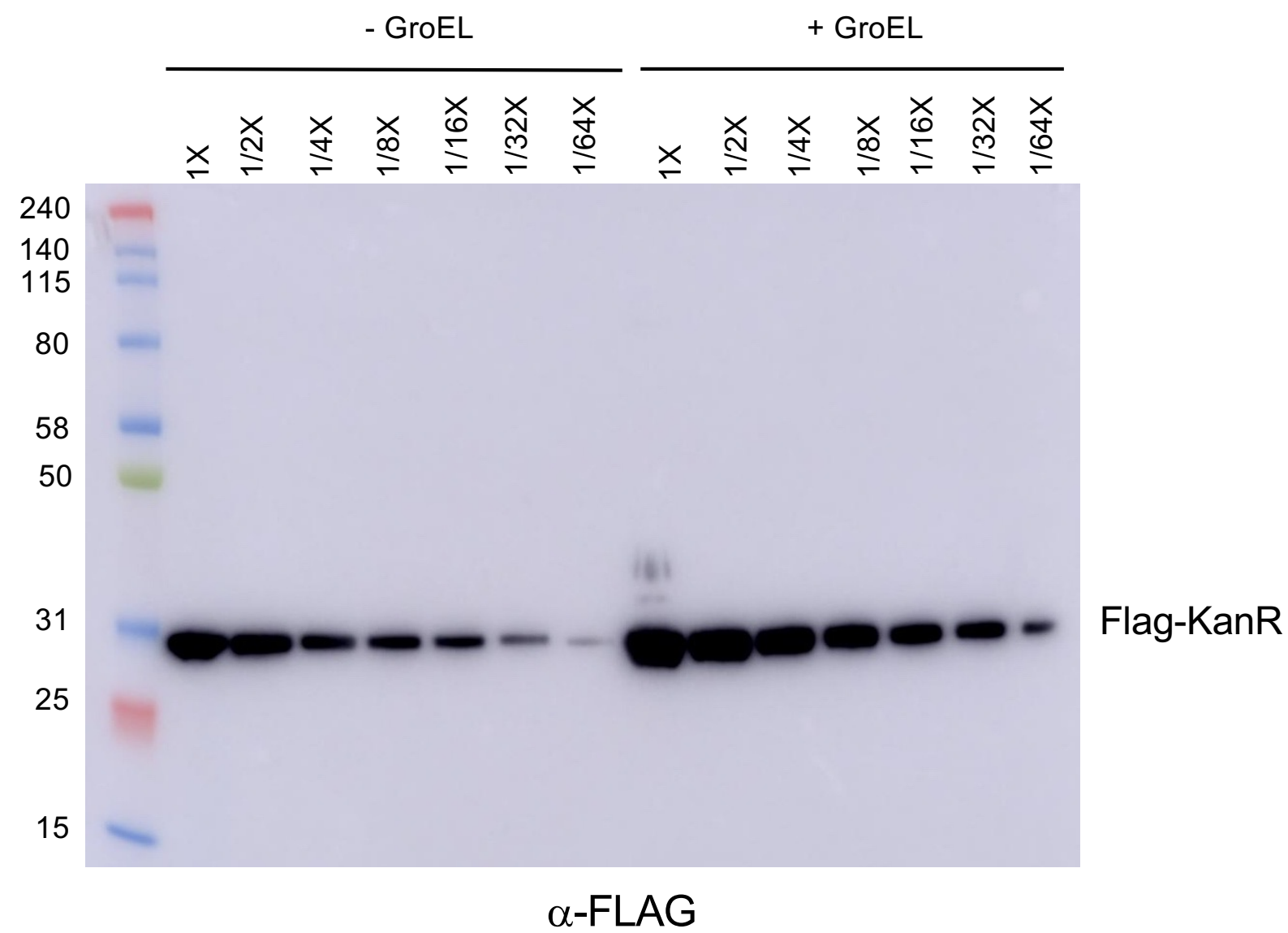

Supplemental Figure 4: GroEL overexpression modestly increases FLAG-KanR protein levels. Comparison of various dilutions of cellular lysates of FLAG-KanR with either the empty vector (-GroEL) or GroEL overexpression (+GroEL) by western blot. Lysate dilution factors are above each well. To the left of the gel, molecular weights (kDa) are shown.
